# Effectiveness of a Per-Meal Protein Prescription and Nutrition Education with versus without Diet Coaching on Dietary Protein Intake and Muscle Health in Middle-Aged Women

**DOI:** 10.3390/nu14020375

**Published:** 2022-01-16

**Authors:** Kelley L. Jackson, Sareen S. Gropper, Dennis Hunt, Deborah D’Avolio, David Newman

**Affiliations:** 1 Christine E. Lynn College of Nursing, Florida Atlantic University, 777 Glades Road, Boca Raton, FL 33431, USA; kjackson2016@health.fau.edu (K.L.J.); ddavolio@health.fau.edu (D.D.); dnewman@health.fau.edu (D.N.); 2 Department of Rehabilitation Sciences, Florida Gulf Coast University, 10501 FGCU Blvd S, Fort Myers, FL 33965, USA; dhunt@fgcu.edu

**Keywords:** protein intake, protein prescription, nutrition education, diet coaching, muscle health

## Abstract

Sufficient dietary protein intake is vital to maintaining muscle health with aging. Yet protein intake among adults is often inadequate. This study’s main objective was to examine the impact of nutrition education (NE) and a per-meal protein prescription (PRx) with versus without diet coaching on protein intake. A secondary objective examined its effects on muscle health. Participants included 53 women, age 45–64 years. All participants received NE and PRx; those randomized to coached-group received 10-weeks of diet coaching. Assessments included: protein intake at baseline, weeks 4 and 12 and muscle health (muscle mass, grip strength, five-chair rise test, 4 mgait speed test). The Chi-square test examined percentages of participants meeting PRx between groups. Repeated measures analysis of variance assessed within group and intervention effects on protein intake and muscle health parameters. Protein intake (g/kg body weight) increased (*p* < 0.001): not-coached (*n* = 28) 0.8 ± 0.2 to 1.2 ± 0.3 and coached (*n* = 25) 1.0 ± 0.2 to 1.4 ± 0.3 with no significant difference between groups. A greater percentage of coached-group participants met (*p* = 0.04) breakfast (72%) and met (*p* < 0.001) three-meal (76%) PRx versus not-coached participants (25% and 53%, respectively). Participants in both groups exhibited significantly (*p* < 0.001) improved times for the five-chair rise test and 4 mgait speed test. Diet coaching in conjunction with a PRx and NE should be considered to assist individuals in improving protein intake through self-selection of protein-rich foods.

## 1. Introduction

Sufficient dietary protein intake is critical to the maintenance of muscle mass, strength, and physical function (i.e., muscle health) or attenuating losses of muscle health that commonly occur with aging. Yet, protein intake among adults is often inadequate [1,2,3,4,5,6,7,8]. National Health and Nutrition Examination Surveys 2005–2014 data from a group of 11,680 adults reported an inadequate protein intake (less than the recommended dietary allowance (RDA) of 0.8 g/kg body weight) among 45% of females and 31% of males aged 51–60 years and among 48% of females and 37% of males aged 61–70 years [4,6]. The percentages rose even higher for adults ≥ 71 years [4,6]. Moreover, a meta-analysis across cohorts residing in Canada, the Netherlands, and the United Kingdom also documented inadequate protein intakes (below the RDA) among older adults living independently, with a higher overall pooled prevalence in women (23.6%) than men (18.8%) [8]. Further, when 1.2 g protein/kg body weight (representing newer recommendations) was used as a cut-off value, the percentage of older adults with inadequate intake ranged from 65–76% [8]. Possible reasons for the insufficient protein intakes include, for example, age-associated reductions in appetite, lack of knowledge about protein recommendations and quantities of food needed to meet recommendations, chewing difficulties, dysphagia, food intolerances, food preparation limitations, skipping meals, and limited financial resources, among others [4].

While the RDA (which was determined based largely on nitrogen balance studies dating back to 1940s and did not rely on indicators such as muscle health) has remained for decades at 0.8 g protein/kg body weight/day for adults, a growing body of research studies have indicated that protein intakes above the RDA, especially for adults > 40 years, are needed to increase muscle protein synthesis and attenuate losses in muscle health (and thus lessen the risk of sarcopenia and its associated adverse consequences) [9,10,11,12,13,14,15,16,17,18,19,20,21,22,23,24]. As a result of these and other studies demonstrating that improved muscle health becomes possible with higher protein intakes, recommendations from expert panels suggest protein intakes of 1.0 to 1.2 g/kg body weight/day for healthy middle- and older-aged adults, and intakes above 1.2 g/kg body weight for adults with chronic and acute medical conditions [23,25,26,27,28,29]. Additional research promotes the consumption of meals providing 20–35 g of high-quality protein (or about 0.4 g protein/kg body weight/meal) to overcome postprandial anabolic resistance and more robustly stimulate muscle protein synthesis [22,23,26,27,28,29,30,31,32,33]. Uneven/skewed protein intake patterns are typical with adults of all ages consuming the majority of dietary protein at dinner (i.e., the evening meal) and the least at breakfast [1,5,34].

Increases in protein intake among adults with inadequate consumption have been shown to positively impact muscle health. Yet, the provision of supplements (primarily as powders containing casein, whey, soy or free amino acids) has been the main approach used in studies evaluating the effects of protein on muscle health [10,12,15,19,23,35]. Only a few studies have examined increasing protein intake by providing herb/spice packets to adults in order to increase egg and protein consumption [36], using ready-made meals [37], and by adding ricotta cheese to the diet [38]. Another approach that has been used successfully to help adults change dietary behaviors associated with chronic diseases (such as type 2 diabetes and heart disease) is diet coaching [39,40,41,42]. Only one study, a single-arm pilot investigation, has assessed its use in improving protein consumption but this was examined in older adults [3]. Absent from the literature, but fulfilled from this study, is an examination of the impact of a per-meal protein prescription and nutrition education that utilized diet coaching in improving protein intake among middle-aged women. Such efforts are important given: (a) protein consumption is often inadequate in middle-aged women; (b) women typically have less muscle mass than men, and (c) the multitude of adverse effects associated with sarcopenia. This study’s primary objective was to evaluate the impact of nutrition education and an individualized per-meal protein prescription with and without diet coaching on protein intake in middle-aged women. A secondary objective investigated the impact of the changes in protein intake on muscle health (i.e., mass, strength, and physical function).

## 2. Materials and Methods

Participants were recruited by oral announcements, fliers, and word of mouth in two eastern South Florida urban communities. Participant inclusion criteria were female, 45–64 years of age, self-reported as healthy, English speaking/reading, able to communicate by telephone, willing to consume protein-containing animal products and make changes to their diet. Exclusion criteria included: self-reported as having renal disease, current or past history of a diagnosed eating disorder, internal cardiac pacemaker, vegan dietary practices, or usual meal-associated dietary protein intake ≥1.2 g protein/kg body weight/day. The University’s Institutional Review Board for the Protection of Human Subjects in Research approved the study. The study is registered at clinicaltrials.gov as NCT04660851.

### 2.1. Assessments

Sociodemographic, Anthropometric, and Dietary. Sociodemographic information (age and education) was gathered at baseline. Height was determined using a height rod at baseline. Weight was measured using an electronic scale (Healthometer, Model 349KLX, McCook, IL, USA) at baseline and 12 weeks. Body mass index (BMI, in kg/m^2^) was calculated from the height and weight measurements.

Intakes of energy, protein, carbohydrate, and fat were assessed at baseline, 12 weeks as well as at week 4 utilizing three (two weekdays, a weekend day) 24-h diet recalls at each time point. Dietary recalls were collected using multiple-pass dietary recall methodology. The recalls were analyzed using diet analysis software (ESHA, Salem, OR, USA) with over 129,000 foods in its database [43,44]. The diet analysis software includes findings from the United States Department of Agriculture Standard Reference database (used for most food composition databases) and from restaurants, food manufacturers, and other sources [44].

Mean protein intake at meals, obtained from the analysis of the three 24-h dietary recalls collected at baseline, was used to determine if participants met the initial study criteria (i.e., protein intake of ≤1.2 g protein/kg body weight/day). Each participant’s protein intake (g/kg body weight) was also compared to intake at weeks 4 and 12, the protein RDA, and the protein requirement (0.66 g protein/kg body weight) [6]. The macronutrient contents of the diet (as a percentage of energy intake) and the percentage of protein ingested from animal and plant sources were also calculated at baseline and 12 weeks.

Muscle Mass. Skeletal muscle mass was assessed via bioelectrical impedance analysis (BIA) (RJL Systems BIA-Quantum IV Analyzer^®^, Clinton Township, MI, USA) at baseline and 12 weeks. Electrodes were attached to the right hand and foot of participants positioned in a supine position on a flat surface. Skeletal muscle mass (whole body) was calculated using the resistance value (ohms) obtained from the BIA and the prediction equation of Janssen, Heymsfield, and Ross [45]. This equation was previously developed and cross-validated against magnetic resonance imaging measures of whole-body muscle mass in a sample of 269 adults, 18 to 86 years and with BMIs from 16 to 48 kg/m^2^ [17].

Skeletal muscle index (SMI) was calculated for each participant (baseline and 12 weeks) by dividing skeletal muscle mass (kg) by body weight (kg) and multiplying by 100 [45]. SMI adjusts for stature and non-skeletal muscle tissues (fat, organ, and bone mass). Each participant’s SMI value was then classified as normal or sarcopenic based on cut-off values established from the Third National Health and Nutrition Examination Survey (NHANES III) data [45]. A SMI was considered normal if it was greater than one standard deviation above the sex-specific mean based on a sample of over 6000 young adults (aged 18 to 39 years) from the NHANES III data [45]. SMI cutoff values and their interpretation for women are: >28% “normal”, between 22% and 28% indicative of Class 1 sarcopenia, and <22% indicative of Class II sarcopenia [45].

Muscle Strength and Function. Skeletal muscle strength and function tests were completed at baseline and 12 weeks. These tests included: dominant-hand grip strength (Jamar dynamometer, Performance Health, Cedarburg, WI, USA), timed 4-m (4 m) gait speed test, and timed five-chair rise test [14,46,47,48]. The 4 m gait speed, five-chair rise, and grip strength assessments were each completed three times (after an initial trial); the average was used for statistical analyses. Grip strength, five-chair rise test, and 4 m gait speed measurements were also compared with age and gender-specific cut-off values (grip strength < 16 kg, five-chair rise times > 15 s, and gait speed ≤ 0.8 m/s) to assess risk for sarcopenia [14,49,50].

### 2.2. Procedures

One week after the collection of baseline data, all participants met individually with a registered dietitian/nutritionist (RDN) and were provided with an individualized per-meal protein prescription (0.4 g protein/kg body weight/meal with adjusted body weight used for individuals with a BMI > 28 kg/m^2^), and nutrition education (verbally and written) focusing on protein-containing food sources and needed food portion sizes to meet each individual’s protein prescription. The provided nutrition education was scripted to ensure fidelity (with the exception of portion size information which was individualized based on the protein prescription). Participants were then randomized to the “with” diet coaching (coached) or “without” diet coaching (not-coached) group.

Coached-group participants received weekly diet coaching for 10 weeks which focused on assisting participants in increasing their protein-rich food selections at meals. Coaching was conducted by virtual appointments by a nurse practitioner and was based on the theory of integrative nurse coaching [51,52]. All participants were instructed at baseline to not change physical activity (including type, duration, and intensity) nor to try to gain/lose weight. At 12 weeks, participants were requestioned on the maintenance of physical activity routines and reassessed for weight, dietary intake, and muscle health (i.e., muscle mass, strength, and function) using the same procedures utilized at baseline. Nutrient intake analysis from food recalls were conducted blindly without knowledge of the participant’s group assignment.

### 2.3. Statistical Analyses

All statistical analyses were conducted using SPSS version 28.0 (SPSS, Inc., Chicago, IL, USA). Using the G*power program with the following input parameters: two groups, two measurements, alpha 0.05, and power 0.80 for a repeated measures analysis of variance between factors, the total sample size needed was 46 (GPower 3.1). A total of 54 participants were recruited to allow for a possible attrition rate of 15%. Descriptive statistics were used to illustrate gender, race/ethic group, and years of education. Independent sample *t*-tests were used to examine differences at baseline between the coached and not-coached groups for age, height, weight, body mass index, skeletal muscle mass, skeletal muscle index, grip strength, five-chair rise times, and 4 m walk times.

A Chi-square test of independence was used to examine the percentage of participants in the coached versus non-coached group who met the protein prescription at each meal and the percentage of participants in each group who met the total 3-meal protein prescription. The 2 × 2 Chi-square test of independence computations produces χ^2^ with use of SPSS 28.0 and tests for a relationship between two categorical variables (goal met and group). The per-meal protein prescription goal was ≥0.4g protein/kg body weight, and the 3-meal protein prescription goal was ≥1.2g protein/kg body weight. The odds ratios (ORs) generated by the Chi-square were also reported.

Repeated measures analyses of variance were used to test both the overall (within groups) and intervention effect (between groups) of coaching on the following dependent variables: energy and macronutrient intakes, muscle mass, muscle strength, and muscle function between two group means (coached vs. not- coached) over time. This technique controls for the violation of independence of measurement assumptions that occurs when conducting repeated measures analyses and provides both within group and between group changes over time. All of these tests were conducted with a statistical significance level of 0.05 and were tested using directional 1-tailed hypotheses. The analysis of variance computations produced an F value and η^2^ eta squared. The F value is the ratio of the difference between group means over time and η^2^ reflects proportion of the unique variance being accounted for.

## 3. Results

Fifty-four women, who met study criteria, enrolled; however, one participant withdrew for non-study associated reasons. The remaining 53 participants (98% retention) completed the study, 25 in the coached group and 28 in the not-coached group. Participants in the coached group attended a mean of 8.6 coaching sessions (out of 10); 64% of participants in the coached group attended at least nine sessions. Mean age, height, weight, and body mass index of the participants at baseline did not statistically significantly differ between the two groups (Table 1).

The majority of participants in both groups were non-Hispanic white (not-coached 75%, coached 84%) followed by African American (not-coached 14%, coached 8%) with 4-year college degree plus some post-college education (not-coached 43%, coached 36%) followed by 4-year college degree (not- coached 36%, coached 32%). No statistically significant differences in education or race were found between the groups.

### 3.1. Anthropometric Findings

At the end of the study, weight and BMI did not significantly differ between participants in the not-coached (68.0 ± 11.3 kg; 25.2 ± 3.7 kg/m^2^) versus the coached (64.3 ± 12.1 kg; 24.3 ± 4.1 kg/m^2^) group. However, the mean weight of the coached group participants slightly but significantly (*p* = 0.002) increased from baseline to the end of the study (63.4 ± 11.9 to 64.3 ± 12.1 kg, respectively).

### 3.2. Dietary Intake Findings

Baseline. No significant differences in protein intake at each meal were found between the two groups (g protein or g protein/kg body weight) (Table 2) at baseline. However, three-meal protein intake, expressed as g/kg body weight, was significantly (*p* = 0.01) greater in the coached group (0.9 ± 0.2) versus the not-coached (0.7 ± 0.2) group. Three-meal protein intake averaged 53.8 ± 14.8 g per day in the coached group and 49.0 ± 13.9 g per day in the not-coached group with no significant difference between groups. At baseline, 45% of participants (four participants in the coached (16%) and eight participants in the not-coached group (29%) consumed less than the RDA for protein.

**Figure 1:**
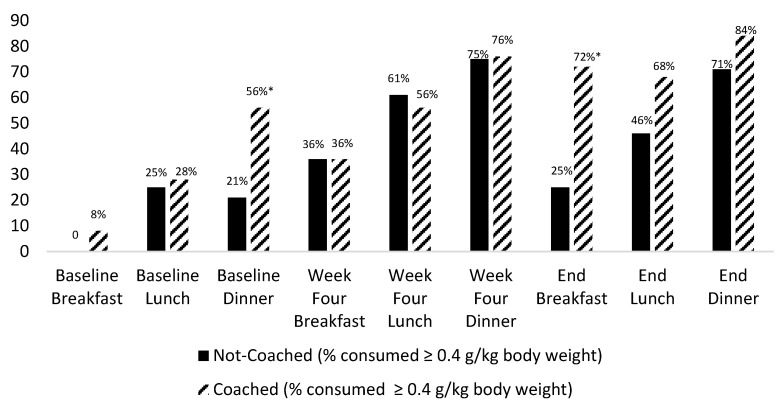
Percentage of participants meeting protein prescription at meals by group at baseline, 4 and 12 weeks (end). * Significant difference coached versus not-coached group participants.

Week 4. The mean protein intake did not significantly differ between the two groups at breakfast, lunch, or dinner at Week 4. Similarly, the percentage of participants in each group who met their protein prescription at each of the meals also did not differ at Week 4 (see Figure 1).

Week 12. At the end of the 12-week study, three-meal protein intake (expressed g/kg body weight) significantly (*p* < 0.001) increased from baseline in both groups. Specifically, in the coached group, protein intake increased from 0.9 ± 0.2 g/kg body weight at baseline to 1.3 ± 0.2 g/kg body weight at 12 weeks and in the not-coached group from 0.7 ± 0.2 at baseline to 1.2 ± 0.3 g/kg body weight at 12 weeks. There was also a significant change in three-meal protein intake (g/day) for both the coached and not-coached groups, with the not-coached group improving from 49.0 ± 13.9 g (baseline) to 80.0 ± 20.4 g (week 4) to 77.4 ± 16.4 g (week 12), and with the coached group improving from 53.8 ± 14.8 g (baseline) to 81.2 ± 16.0 g (week 4) to 84.2 ± 14.5 g (week 12) [V = 0.76, F(2, 50) = 80.5, *p* < *0*.001, η^2^ = 0.763]. There was no statistically significant intervention by time interaction effect [V = 0.03, F(2, 50) = 0.68, *p* = *0*.513, η^2^ = 0.026].

When examining protein intake (g/kg body weight) at each meal at 12 weeks, intake at breakfast (but not lunch and dinner) was significantly (*p* = 0.004) greater in the coached group (0.4 ± 0.1 g/kg body weight) than the not-coached group (0.3 ± 0.1 g/kg body weight). Further, at 12 weeks none of the participants in the coached group versus three participants (11%) in the not-coached group consumed less the RDA for protein.

**Figure 2:**
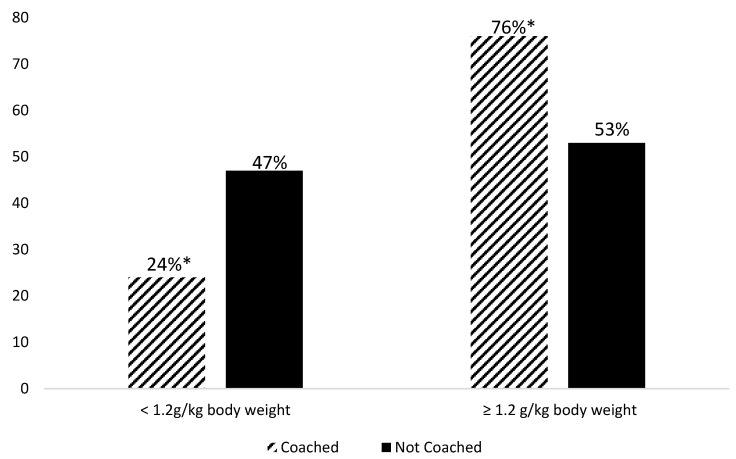
Percentage of participants meeting the three-meal protein prescription by group. * Significantly different (*p* = 0.04) coached versus not coached.

Figure 1 reports the percentages of the participants in each group meeting the per-meal protein prescription at baseline and weeks 4 and 12. At baseline, a significant difference was evident between the groups at only dinner, with a significantly greater percentage of coached group participants (56%) meeting the per-meal protein prescription than the not-coached group participants (21%). The effect of the coaching was clear at 12 weeks with 72% of participants in the coached group meeting the protein prescription at breakfast versus only 25% of participants in the not-coached group (*p* < 0.001). The participants who received diet coaching were 7.7 times more likely (OR χ^2^ (1) 11.71, *p* < 0.001, *Phi* 0.47) to meet their protein prescription at breakfast than those in the not-coached group. Moreover, at 12 weeks, the percentage of participants in the coached group (76%) who met the three-meal protein prescription, i.e., ≥1.2 g protein/kg body weight per day, was significantly (*p* = 0.04) greater than those in the not-coach group (53%) (Figure 2). The coached participants were 2.7 times more likely (OR χ^2^ (1) 2.89 *p* = 0.04, *Phi* 0.23) to meet their protein prescription than the not-coached group participants.

The overall pattern exhibited by the two groups (as shown in Figure 1) is also noteworthy. The percentage of not-coached participants who met the protein prescription at each meal increased between baseline and week 4 but then declined again at week 12: breakfast (0% to 36% to 25%, respectively), lunch (25% to 61% to 46%, respectively), and dinner (21% to 75% to 71%, respectively). This pattern is in contrast to what was observed (i.e., continued improvements at each meal) among participants in the coached group at baseline, week 4, and week 12: breakfast (8% to 36% to 72%, respectively), lunch (28% to 56% to 68%, respectively), and dinner (56% to 76% to 84%, respectively). An evaluation of the number of participants who met their protein prescription at each meal at 12 weeks showed that 12 of the 25 (48%) participants in the coached group met this goal versus only 5 of 28 (18%) participants in the not coached group.

Energy intake did not significantly differ between groups at baseline (not-coached 1397 ± 254 kcal; coached 1398 ± 245 kcal) or at 12 weeks (not-coached 1363 ± 413 kcal; coached 1323 ± 427 kcal). No significant differences were found within groups between baseline and 12-week energy intakes. Protein intake as a percentage of energy rose significantly (*p* < 0.001) within the not-coached group (16% at baseline to 25% at week 12) and the coached group (18% at baseline to 27% at week 12) but did not differ significantly between groups. At 12 weeks, 75% of protein intake was from animal sources in both groups; this compares to 67% of dietary protein intake from animal sources in both groups at baseline. Fat intake represented 40% of energy at both baseline and 12 weeks in the not-coached group and 39% of energy at baseline and 37% of energy at 12 weeks in the coached groups. As a percentage of energy, carbohydrate intake comprised 40% at baseline and 37% at 12 weeks in the not-coached group, and 41% at baseline and 37% at 12 weeks in the not-coached group.

### 3.3. Muscle Mass, Strength and Function Findings

At baseline, no significant differences were found between the two groups for skeletal muscle mass, SMI, and times to complete the five-chair rise test and 4 m gait speed test; however, grip strength was significantly greater in the not-coached versus the coached group (Table 3). At 12 weeks, skeletal muscle mass and SMI did not significantly differ from baseline in either group, and did not significantly differ between groups. Grip strength also did not significantly differ between baseline and week 12 in either group; however, grip strength remained significantly greater in the not-coached versus the coached group. In contrast, at 12 weeks, participants in both groups exhibited significantly (*p* < 0.001) improved times for the five-chair rise test and the 4 m gait speed test (Table 3).

### 3.4. Risk for Sarcopenia

When compared to cut-off points indicating risk for sarcopenia at baseline one participant (2%) in the coached group had a gait speed ≤0.8 m/s and one participant (2%) in the not-coached group had a five-chair rise test >15 s. By 12 weeks, both participants exhibited improved times and were no longer classified at risk for sarcopenia. Based on baseline SMI, five coached-group and five not-coached group participants (19% of total sample) had a SMI of 22–28% consistent with Class I sarcopenia, and one not-coached group participant (2% of total sample) had a SMI classified as Class II sarcopenia. Thus, at baseline, 21% of this group had a SMI indicative of sarcopenia. At 12 weeks, no changes occurred with the exception that one of the five not-coached group participants went from Class I to Class II sarcopenia and the not-coached group participant with Class II sarcopenia was reclassified with Class I sarcopenia.

## 4. Discussion

This study demonstrated the effectiveness of diet coaching (along with a per-meal protein prescription and nutrition education) in assisting middle-aged women with self-selection of protein-rich foods to promote behavior change (improve their protein intake). Yet, while both the coached and the not-coached groups exhibited significant increases in dietary protein intake, a significantly greater percentage of coached-group participants met the daily (three-meal) protein prescription (≥1.2 g protein/kg body weight/day) and the breakfast protein prescription than the not-coached group participants. The odds ratios for these tests suggest a substantial difference between the coach-group and not-coached group participants in the percent of participants meeting the protein prescription at breakfast and at three meals and contrast with prior research indicating a medium effect size in protein intake. It also highlights the value in assessing the numbers (percentages) of participants meeting recommendations versus assessing only mean protein intake of the groups. This study’s findings suggest that the use of the per-meal protein prescription, nutrition education, and diet coaching facilitated a daily protein intake that better meets higher protein recommendations [23,25,26,27,28,29]. It also facilitated the consumption of a more even distribution of protein among meals.

This study intervention is novel in that it directly worked with participants via coaching to promote behavior change, i.e., increased protein consumption via self-selection of protein-rich food sources. This finding is consistent with the scientific literature examining the effectiveness of diet coaching. Coaching intervention studies, including systematic reviews, have typically demonstrated positive outcomes, with desired effects shown in one or more dietary behaviors [39,41,42]. This study extends the effectiveness of diet coaching to middle-aged women with suboptimal protein intake. The exact number of coaching sessions and time frame that are needed to effectively promote behavior change is unclear. A rather large timeframe range, from about two weeks to two years, is reported in a review of the coaching literature [42]. This present study demonstrated positive behavior change as the result of nutrition education and a per-meal protein prescription with and without diet coaching at study week 4. However, at week 12, dietary behaviors in the not-coached group had reverted back towards those exhibited at baseline. These findings suggest that while nutrition education and a protein prescription may be valuable in initiating and maintaining behavior change over a 4-week period, the effectiveness of behavior change without the coaching intervention wanes sometime between 4 and 12 weeks. Future investigations are needed to the determine the frequency and timing of diet coaching that most benefits behavior change.

Muscle Health. The observed significant increases in total protein intake shown in both the coached and not-coached groups are thought to have contributed to the significant improvements observed in lower leg muscle strength and function (demonstrated by the shorter times needed to complete the five-chair rise and 4 m gait tests). These beneficial findings may be related to enhanced maintenance and repair of existing muscle fibers secondary to the higher protein intakes [23]. However, in addition, greater familiarity with the testing/assessment protocols may also have contributed. The inclusion of a control group receiving no protein prescription or nutrition education would be beneficial in future studies, although it was assumed no changes in protein intake or muscle health would have been found without the intervention. Improved lower-body muscle strength and function have been documented in other studies that have included mostly older-aged adults provided with protein supplements [10,22,30]. The findings that individuals in both the coached and not-coached groups exhibited improvements in 4 m gait speed and five-chair rise times also suggests that the increase in overall daily dietary protein maybe of more significance than meeting a prescribed per-meal quantity of protein. Whether or not a more even distribution of dietary protein among meals is of benefit versus ensuring the consumption an adequate total daily protein intake remains unclear [21,25,33,53].

Grip strength, a validated method of assessing upper limb muscle strength, did not significantly differ within groups over the 12-weeks, and no study participants had grip strength values indicative of risk of sarcopenia [54]. Other studies have also found no gains in grip strength among older adults provided with protein supplementation [12,22,38].

Neither skeletal muscle mass nor SMI differed significantly within groups over this 12-week study. These findings are consistent with another study in which 47 older women exhibited no significant change in muscle mass after 10 weeks of receiving either high protein (1.2 g/kg body weight) or the protein RDA [55]. In contrast, a few studies have reported significant gains in muscle mass in older adults given supplemental protein. Mitchell and colleagues [21] reported significant increases in trunk and leg muscle mass in 29 older men provided with 1.6 g protein/kg body weight/day (versus the RDA) over a 10-week period. Chanet and coworkers [12] showed significant increases in muscle protein synthesis and leg muscle mass in 24 older men supplemented with 20 g whey protein, 800 IU vitamin D, and 3 g leucine versus a placebo over a 6-week time frame. Similarly, Aleman-Mateo and associates [38] found that 100 healthy older adults provided with 210 g ricotta cheese daily (containing 18 g protein versus a placebo) for 12 weeks exhibited a small but significant increase (0.6 kg) in appendicular skeletal muscle mass. The reason(s) for the lack of significant increases in muscle mass in the present study is not clear. Given the improvements in lower limb muscle mass in these other studies [12,21,38], it is possible that differences may have been identified in the present study had appendicular (versus whole-body) muscle mass been measured. Another possible explanation as to the absence of effects of increased protein intake on muscle mass and grip strength may be related to the engagement of the study participants in resistance/strength training [56], although participants reported no changes in physical activity. A longer study duration or higher protein intakes may also have been needed to have demonstrated significant increases in muscle mass in the middle-aged women participating in the study.

Limitations. This study had several limitations including the relatively small sample size of primarily highly educated, non-Hispanic white middle-aged women dwelling in South Florida. Additional studies examining the impact of diet coaching on protein intake that are of longer duration, that include an additional control group not given a per-meal protein prescription or nutrition education, and that includes larger more diverse groups of women are warranted especially given 45% of the participants were not consuming the RDA for protein. Further, while this study found a 21% prevalence of sarcopenia based on BIA-derived SMI and compared with NHANES III based cut-off values and the work of Janssen, Heymsfield and Ross [45], additional studies should be conducted examining the prevalence of sarcopenia in middle-aged women based on the latest means of identification.

## 5. Conclusions

Increased dietary protein intake was achieved by middle-aged women with the use of nutrition education and a per-meal protein prescription. However, when nutrition education and a per-meal protein prescription were combined with diet coaching, more participants achieved the higher daily protein intake and a more even distribution of protein among meals. While the study’s nutrition education component afforded participants the opportunity to gain knowledge in order to self-select preferred high-quality protein sources to meet their individualized daily protein prescription, the diet coaching enabled a greater percentage of the participants to sustain behavior change after 4 weeks and through the rest of the 12-week study period. Diet coaching should be considered in conjunction with nutrition education and a protein prescription to assist individuals in sustaining behavior change. Both the gained knowledge and the changed dietary behaviors adopted by participants, if sustained long term, are thought to be important to maintain muscle health and to help in the prevention of age-related conditions such as sarcopenia and its associated adverse consequences.

## Figures and Tables

**Table 1:** Age and Anthropometric Characteristics of Participants at Baseline.

Baseline Characteristics	Not-Coached (*n* = 28)		Coached (*n* = 25)			
	Mean	SD	Mean	SD	t(51)	*p*
Age (years)	56.4	4.8	55.1	5.7	0.95	0.35
Height (cm)	164.1	7.3	162.1	6.4	1.35	0.33
Weight (kg)	67.6	11.1	63.4	11.9	1.33	0.19
Body Mass Index (kg/m^2^)	25.1	3.7	24.2	3.9	0.84	0.41

**Table 2:** Protein Intakes of Participants at Meals at Baseline.

Protein Intake	Not-Coached		Coached		
	(*n* = 28)		(*n* = 25)		
	Mean	SD	Mean	SD	*p*
Breakfast					
Protein (g)	9.6	6.3	11.5	6.5	0.29
Protein (g/kg body weight)	0.1	0.1	0.2	0.1	0.17
Lunch					
Protein (g)	18.1	7.1	19.9	7.8	0.38
Protein (g/kg body weight)	0.3	0.1	0.3	0.1	0.10
Dinner					
Protein (g)	21.6	6.9	24.1	6.5	0.18
Protein (g/kg body weight)	0.3	0.1	0.4	0.1	0.06
3-meals					
Protein 3-meals (g)	49.0	13.9	53.8	14.8	0.07
Protein 3-meals (g/kg body weight)	0.7	0.2	0.9	0.2	0.01

**Table 3:** Skeletal Muscle Mass (SMM), Skeletal Muscle Index (SMI), Grip Strength (GS), Five-Chair Rise, and 4-m (4 m) Gait Speed Measures of Participants at Baseline and 12 Weeks.

Outcomes	Not-Coached				Coached				Within Group Change by Time			
	Baseline		12 Weeks		Baseline		12 Weeks		*F*	*Df*	*p*	*n^2^*
	Mean SD		Mean SD		Mean SD		Mean SD					
SMM (kg)	19.8	2.0	19.5	2.1	19.1	2.1	19.0	2.3	0.223	1.50	0.64	0.004
SMI (%)	30.3	4.6	29.7	4.7	30.6	3.6	29.9	3.5	0.018	1.50	0.89	0.000
GS (kg)	25.0	5.4	25.8	5.1	21.5	4.5	21.9	5.2	1.62	1.48	0.21	0.033
5-Chair Rise (s)	9.2	2.8	7.3	2.3	7.9	2.0	6.8	1.8	42.41	1.49	<0.001	0.464
4 m Gait Speed (s)	3.3	0.6	3.0	0.4	3.1	0.5	2.9	0.4	26.68	1.50	<0.001	0.348
**Between Group Change by Time Intervention**												
SMM (kg)									1.13	1.50	0.29	0.022
SMI (%)									0.063	1.50	0.80	0.001
GS (kg)									0.21	1.48	0.32	0.004
5-Chair Rise (s)									3.50	1.50	0.07	0.067
4 m Gait Speed (s)									0.10	1.50	0.38	0.002

Abbreviations: SMM = skeletal muscle mass. SMI = skeletal muscle index. GS = grip strength.

## Data Availability

The data presented in this study are available on request from the corresponding author. They have not yet been entered into a repository.
